# Performance of Repair Mortars Composed of Calcium Sulfoaluminate and Amorphous Calcium Aluminate

**DOI:** 10.3390/ma19020261

**Published:** 2026-01-08

**Authors:** Seungtae Lee, Seho Park

**Affiliations:** Department of Civil Engineering, Kunsan National University, Kunsan 54150, Jeonbuk, Republic of Korea; sekhok88@kunsan.ac.kr

**Keywords:** repair mortar, calcium sulfoaluminate cement, amorphous calcium aluminate cement, mechanical properties, freeze–thaw resistance

## Abstract

Extensive research has addressed concrete deterioration and its countermeasures; however, studies on responsive repair methods and materials remain comparatively limited and less systematic. In this study, six mixtures of repair mortars (RMs) were formulated using aluminate-based binders, specifically calcium sulfoaluminate (CSA) and amorphous calcium aluminate (ACA) cements. The experiment evaluated the mechanical properties and freeze–thaw resistance of these mortars. To accelerate hydration, a controlled amount of anhydrite gypsum was incorporated into each mixture. The fluidity and setting time of fresh RMs were measured, whereas the compressive strength, flexural strength, and ultrasonic pulse velocity (UPV) of hardened RMs were evaluated at 1, 7, and 28 days. In addition, freeze–thaw resistance was assessed as per ASTM C666 by determining the relative dynamic modulus of elasticity. Additionally, the hydration products and microstructural characteristics of paste specimens were qualitatively analyzed. The mechanical performance, including strength and UPV, and freeze–thaw resistance of RMs containing ACA were superior to those of RMs containing CSA. In particular, compared to the CSA-containing specimens exposed to freeze–thaw action were significantly deteriorated, the ACA-containing specimens showed excellent resistance with relatively less cracking and spalling. This may imply that ACA is effective as rapid repair materials for concrete structures in cold regions. Microstructural observations revealed variations in hydration products depending on the aluminate binder employed, which significantly influenced the mechanical and durability properties of the RMs. These results may aid the selection of optimal repair materials for deteriorated concrete structures.

## 1. Introduction

Extensive research has been conducted globally to identify the causes of concrete deterioration and develop effective countermeasures. However, studies focusing on repair methods and materials that proactively address deterioration remain comparatively limited. Among the various repair materials, aluminate-based binders, such as calcium sulfoaluminate (CSA) and amorphous calcium aluminate (ACA) cements, have been extensively investigated for their potential to enhance both the mechanical performance and durability of concrete structures, yielding significant research outcomes to date [1,2].

CSA cement, primarily manufactured from bauxite, limestone, and gypsum, was first developed in the 1960s. Thenceforth, CSA has been widely utilized as a shrinkage-compensating additive and, in some countries, as an expansive binder owing to the expansive properties of ye’elimite (3CaO_3_·Al_2_O_3_·CaSO_4_) present in CSA [3,4]. Owing to its rapid setting, quick strength development, and slight expansion, CSA is particularly well suited for rapid repair applications, such as mitigating microcracks in bridge expansion joints [5,6,7,8]. Research on CSA has addressed the strength, durability, hydration reactions, and hydration modeling of the material [9,10,11]. For example, Coumes et al. [3] demonstrated that hydration of CSA is accelerated by an increased nucleation rate, thereby enhancing early strength development. Additionally, Chen et al. [5] elucidated the correlation between alkalinity in pore solution and AFt formation.

ACA cement, primarily composed of C_12_A_7_ with an alumina content of approximately 40–50%, is characterized by the formation of substantial quantities of needle-like AFt and C_2_AH_8_ during hydration, resulting in rapid setting [12]. When combined with ordinary Portland cement (OPC), the presence of Ca(OH)_2_ moderates the setting rate, thereby enhancing workability. Additionally, the incorporation of anhydrite accelerates hydration, making ACA suitable for shotcrete applications and rapid repair of concrete structures.

In this study, repair mortars (RM) were formulated by partially replacing OPC with CSA and ACA. The mechanical performance of these mortars was evaluated through measurements of fluidity, setting time, mechanical strength, and ultrasonic pulse velocity (UPV). Paste samples containing aluminate-based binders (CSA and ACA) were further analyzed to identify hydration products using X-ray diffraction (XRD) and to investigate microstructural characteristics using scanning electron microscopy (SEM). Furthermore, freeze–thaw resistance tests were conducted to determine the suitability of these RMs for use in cold climates and winter conditions. The experimental results are anticipated to provide valuable insights for selecting the optimal repair materials for deteriorated concrete structures.

## 2. Experimental Program

### 2.1. Materials

**Figure 1:**
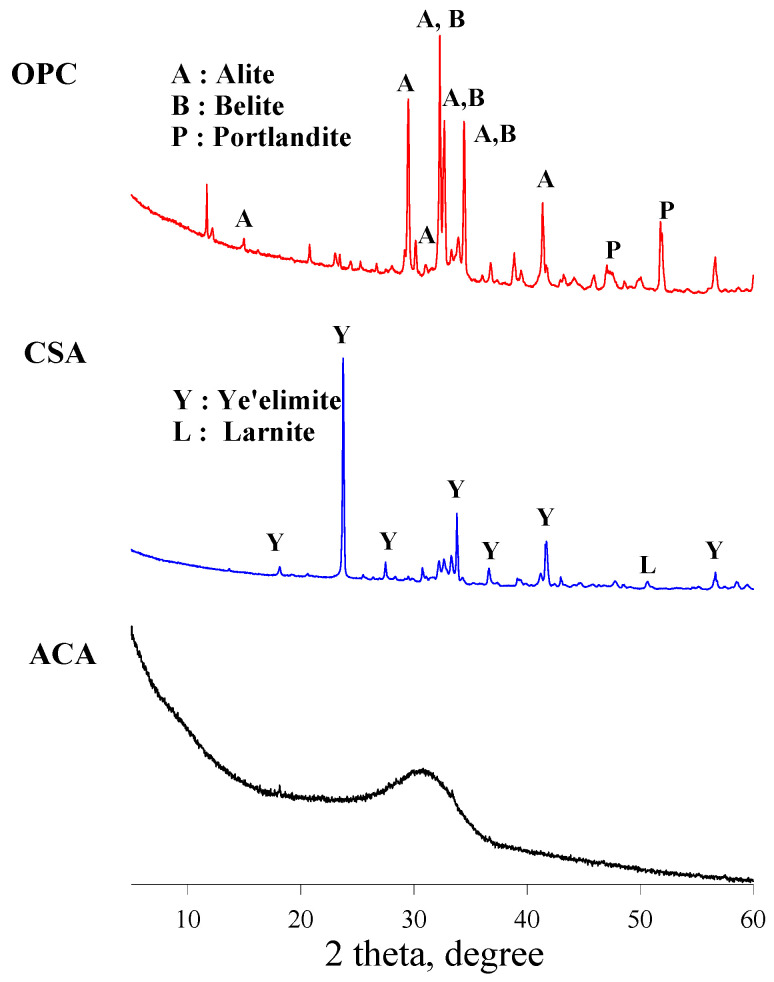
XRD patterns for binders utilized in the study.

**Figure 2:**
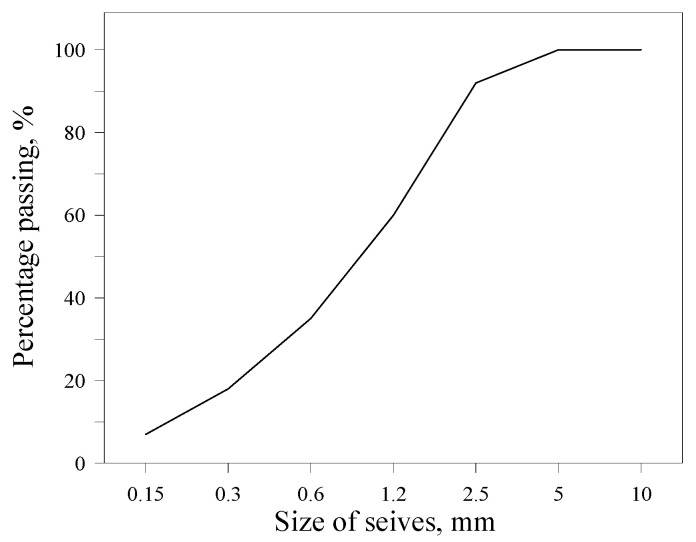
Size grading of fine aggregate.

The binders utilized in this study were OPC conforming to ASTM C150-24 [13], CSA cement, and ACA cement. To accelerate hydration, anhydrite gypsum (AG) was incorporated in varying proportions depending on the mixture. The chemical compositions of the binders are listed in Table 1, and their XRD patterns are shown in Figure 1. As indicated by the XRD results, CSA comprises primarily crystalline ye’elimite, whereas ACA cement is predominantly amorphous C_12_A_7_ [14]. Silica sand was used as a fine aggregate with a fineness modulus of 2.88, a water absorption of 1.14% and a bulk density of 2.64 g/cm^3^. The size grading of fine aggregate is shown in Figure 2.

### 2.2. Mix Proportions

Six RM mixtures were formulated, as detailed in Table 2. The RMC-1 mixture was prepared by substituting 25 wt% of OPC with CSA and AG. In RMC-2, 40 wt% CSA and 30 wt% AG replaced OPC. The RMA-1 mixture incorporated 25 wt% ACA and AG as OPC replacements, whereas RMA-2 utilized 40 wt% ACA and 30 wt% AG. For RMCA-1 mixture, 12.5 wt% CSA, 12.5 wt% ACA, and 25 wt% AG were substituted for OPC, whereas RMCA-2 contained 20 wt% CSA, 20 wt% ACA, and 30 wt% AG. All mixtures maintained a constant water-to-binder ratio (w/b) of 57% to ensure comparable setting and workability. The binder-to-fine aggregate ratio was fixed at 1:2 for all mixes. The mixing odder of the RM mixtures is as follow. First, the binders were mixed together and dry-mixed for 30 s, then fine aggregate was added and dry-mixed for another 30 s. Thereafter, distilled water was added, and additional mixing was performed for a total of 1.5 min.

Additionally, paste samples with the same w/b ratios (57%) as the mortar samples, were prepared for each binder system to facilitate analysis of hydration products and microstructural characteristics through XRD and SEM.

### 2.3. Test Methods

Mortar fluidity was evaluated in accordance with ASTM C1437-20 [15], whereas the setting time was determined following ASTM C807-21 [16].

Mortar specimens were demolded 24 h after casting and subjected to tap water curing at 20 ± 3 °C. Compressive and flexural strengths were evaluated at 1, 7, and 28 days, in accordance with ASTM C109/C109M-21 [17] and C348-21 [18], respectively.

UPV was determined in accordance with ASTM C597-22 [19].

Freeze–thaw resistance was evaluated in accordance with ASTM C666-97 [20]. The dynamic modulus of elasticity was measured every 30 cycles, and the relative dynamic modulus of elasticity (RDME) was calculated using Equation (1) as follows:

(1) $$RDME=\;(\frac{n_{c}}{n_{0}}{)}^{2}\times 100(\%)$$

where $n_{c}\;$ represents the fundamental transverse frequency after c cycles of freeze–thaw (Hz) and $n_{0}\;$ represents the fundamental transverse frequency at 0 cycles of freeze–thaw (Hz).

To evaluate hydration products, paste samples cured under water for 28 days were analyzed using XRD. The measurement conditions were as follows: CuKα radiation with a nickel filter, an operating voltage of 30 kV, a current of 20 mA, a scanning speed of 2°/min, and a 2θ range of 5–60°.

Microstructural observations were conducted on paste samples cured for 1 and 28 days using SEM. Hydration was terminated by immersing the samples in acetone for 6 h, followed by vacuum drying for 24 h. Prior to observation, sample surfaces were coated with platinum. An ultra-high resolution field emission-SEM (SU-8220) was utilized to examine the microstructural features.

## 3. Results and Discussion

### 3.1. Fluidity

**Figure 3:**
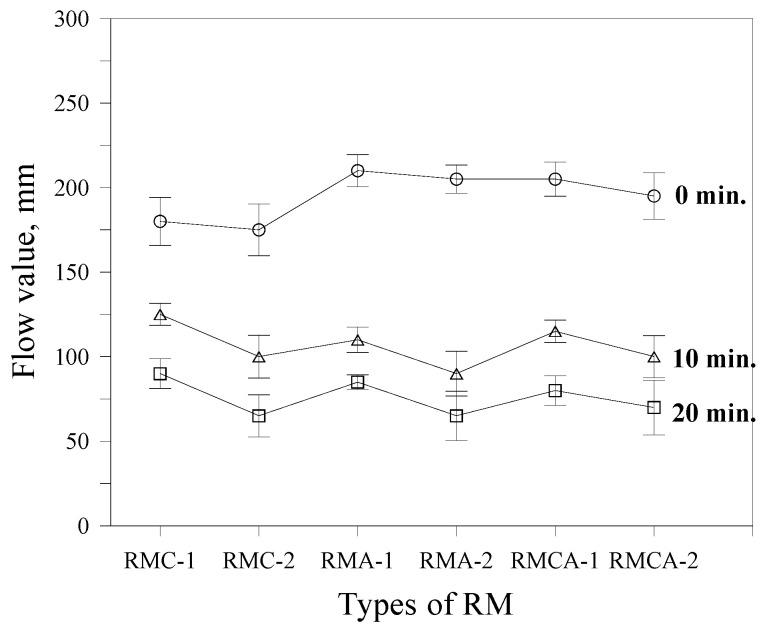
Fluidity of RM.

The variation in fluidity of the RMs is shown in Figure 3. At the initial measurement (0 min), mortars containing ACA (RMA-1 and RMA-2) demonstrate slightly higher flow values compared with those containing CSA (RMC-1 and RMC-2). However, after 10 min, ACA-based mortars experience a more pronounced loss in fluidity, resulting in lower flow values compared with CSA-based RM. Mortars incorporating both CSA and ACA (RMCA-1 and RMCA-2) demonstrate fluidity trends similar to those of ACA mortars, regardless of the elapsed time.

Meanwhile, increasing the content of aluminate-based binders (CSA + ACA) results in reduced fluidity. For example, the flow values of RMA-2 (14% ACA) are 175, 100, and 65 mm at 0, 10, and 20 min, respectively, whereas RMA-1 (8.75% ACA) records 180, 125, and 90 mm over the same time intervals. Similar trends are observed in both RMC and RMCA mortars.

### 3.2. Setting Time

**Figure 4:**
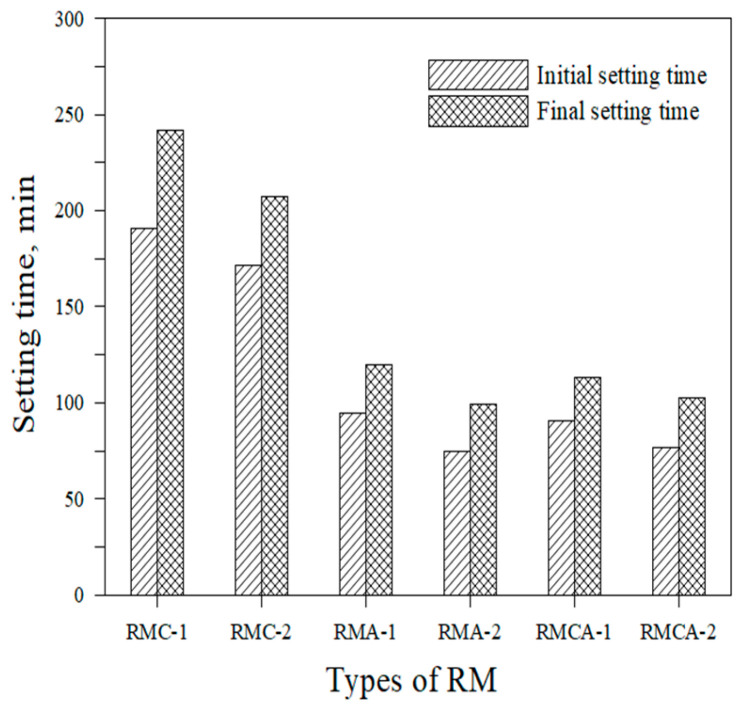
Setting time of RM.

A comparative analysis of the initial and final setting times of the six RMs is shown in Figure 4. RMC mortars display the longest setting times, whereas RMA and RMCA mortars demonstrate relatively shorter and similar setting behaviors. Notably, the setting times of RMA mortars are approximately 50% shorter than those of RMC mortars. This behavior is attributed to the hydration of C_12_A_7_, the principal component of ACA, which rapidly forms C_2_AH_8_ at the early stages of hydration, as represented using Equation (2) [12].

C_12_A_7_ + 51H → 6C_2_AH_8_ + AH_3_ (2)

Increasing the dosage of aluminate-based binders (CSA + ACA) consistently results in shorter setting times, regardless of mortar type.

### 3.3. Compressive and Flexural Strength

**Figure 5:**
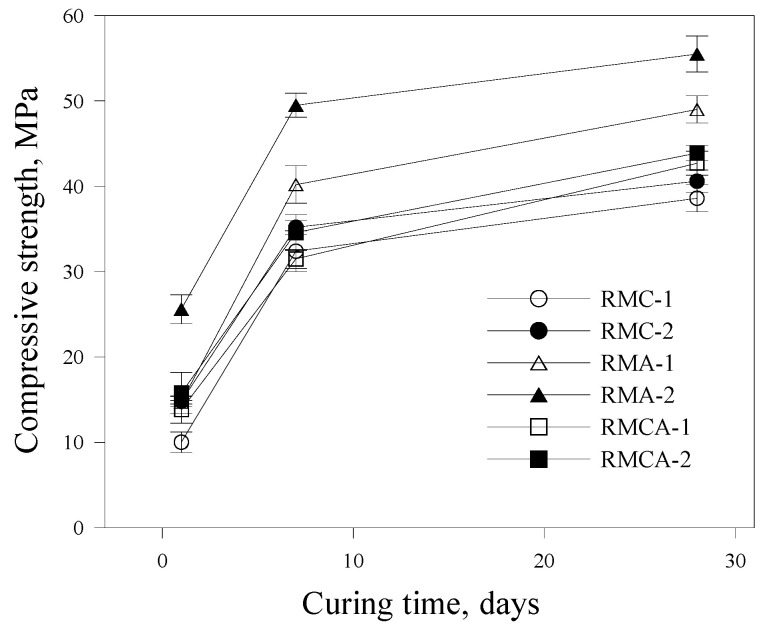
Compressive strength development of RM.

The compressive strength development of the RMs is shown in Figure 5. Compressive strength increases progressively with curing age. CSA mortars (RMC-1 and RMC-2) display lower compressive strengths compared with ACA mortars (RMA-1 and RMA-2) from the earliest ages. After 1 day of curing, RMA-1 and RMA-2 achieve compressive strengths of approximately 14.7 and 25.6 MPa, respectively, whereas RMC-1 and RMC-2 reach only 10.0 and 14.8 MPa, respectively. The differences become more pronounced after 28 days of curing. The superior performance of ACA mortars is attributed to the formation of aluminate hydrates during hydration, which enhance early strength development [14,21].

Furthermore, the effect of binder dosage is evident. RMA-2, containing a higher proportion of ACA, achieves compressive strengths of approximately 25.6, 49.5, and 55.5 MPa at 1, 7, and 28 days, respectively, whereas RMA-1 records lower values of 14.7, 40.2, and 49.0 MPa. In contrast, variations in CSA dosage result in less significant changes in compressive strength.

**Figure 6:**
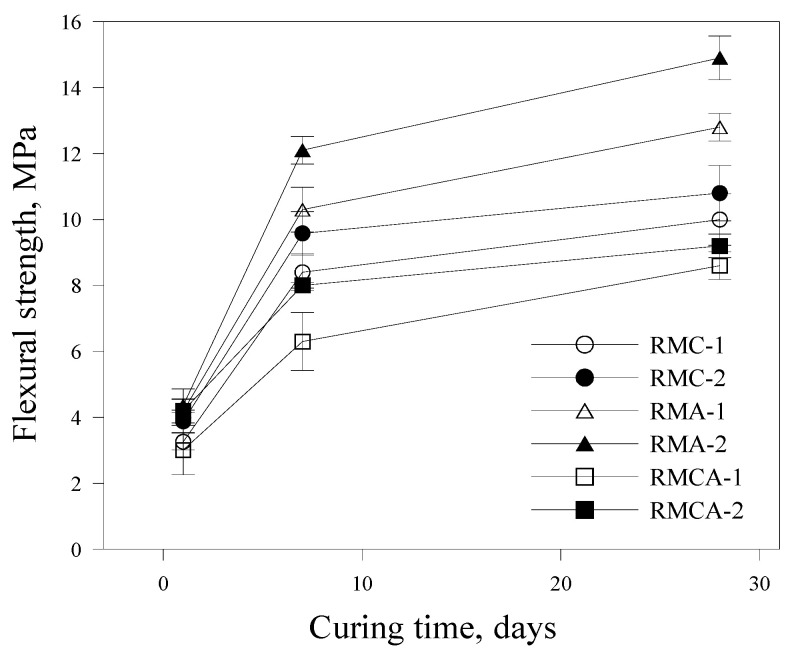
Flexural strength development of RM.

The results of flexural strength are shown in Figure 6. The flexural performance is influenced by the type of aluminate binder. After 1 day of curing, RMA-2 achieves a flexural strength of approximately 4.3 MPa, compared with 3.8 and 4.2 MPa for RMC-2 and RMCA-2, respectively. After 28 days of curing, the differences are more pronounced: RMA-2 reaches approximately 14.9 MPa, whereas RMC-2 and RMCA-2 record values of 10.8 and 9.2 MPa. These findings validate the superior flexural resistance of ACA mortars compared with that of CSA mortars. However, unlike compressive strength, RMC mortars display slightly higher flexural strength compared with that of RMCA mortars.

Both compressive and flexural strengths are significantly influenced by the dosage of aluminate-based binders, with higher binder content resulting in increased strengths. This enhancement is attributed to variations in the type and quantity of hydration products formed in the RM matrix [22].

**Figure 7:**
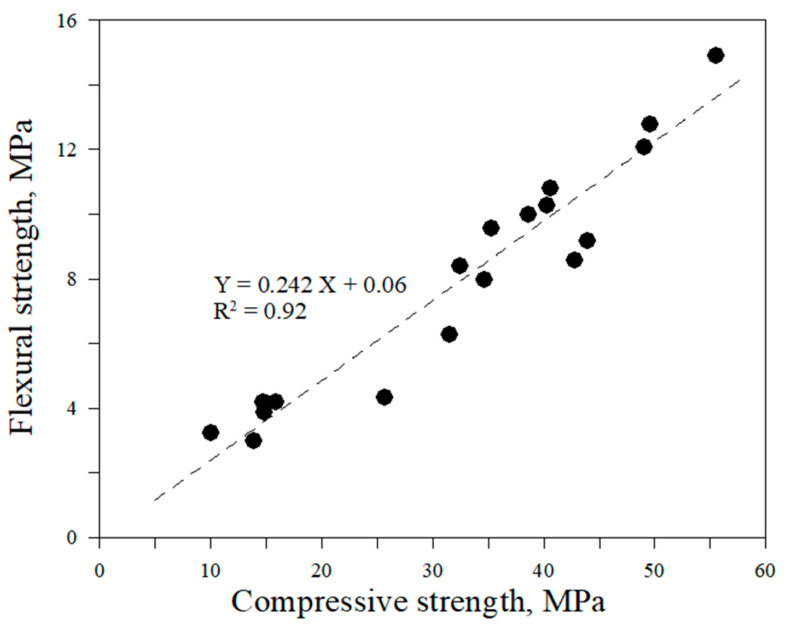
Relationship between compressive and flexural strength of RM.

Compressive and flexural strengths are strongly correlated, with an R^2^ value of 0.92, as shown in Figure 7.

### 3.4. Ultrasonic Pulse Velocity (UPV)

**Figure 8:**
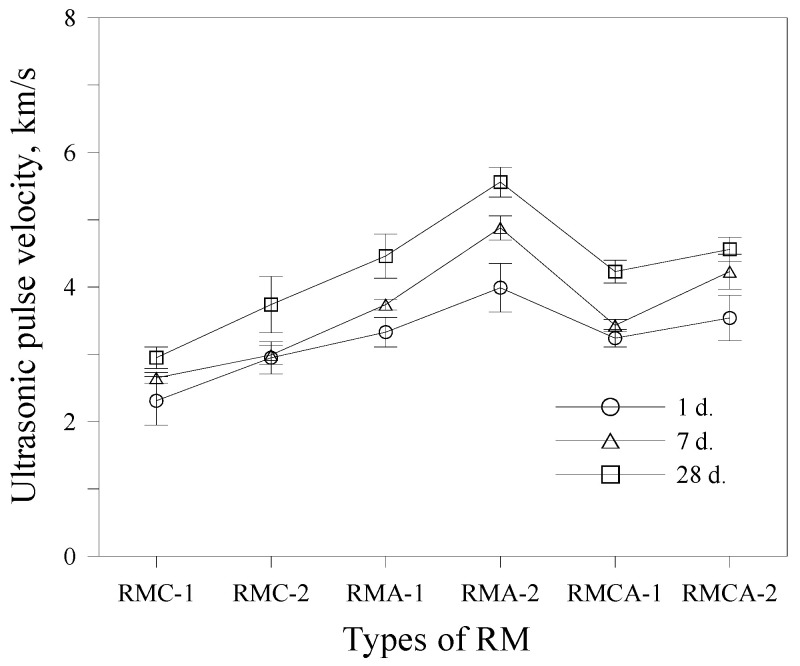
Ultrasonic pulse velocity of RM.

The UPV results of the RMs at various curing ages are shown in Figure 8. UPV values increase with curing age across all mixtures. At each age, mortars incorporating ACA consistently display higher UPV values compared with those containing CSA, which demonstrate relatively lower values. An increase in aluminate-based binder content leads to higher UPV, with this effect being more pronounced in ACA mortars. For example, after 1 day of curing, the UPV of RMC-2 is 2.95 km/s, whereas that of RMA-2 is 3.99 km/s. After 28 days of curing, RMC-2 and RMA-2 reach approximately 3.74 km/s and 5.56 km/s, respectively.

Mortars containing both CSA and ACA (RMCA series) display slightly higher UPV values compared with CSA mortars across all curing ages. Overall, the UPV trends closely match those observed in compressive strength.

### 3.5. Freeze–Thaw Resistance

The freeze–thaw resistance of the six RM mixtures was evaluated through exposure tests conducted in accordance with ASTM C666-97 [20]. The RDME was measured every 30 cycles to evaluate the resistance of each mixture to freeze–thaw damage.

**Figure 9:**
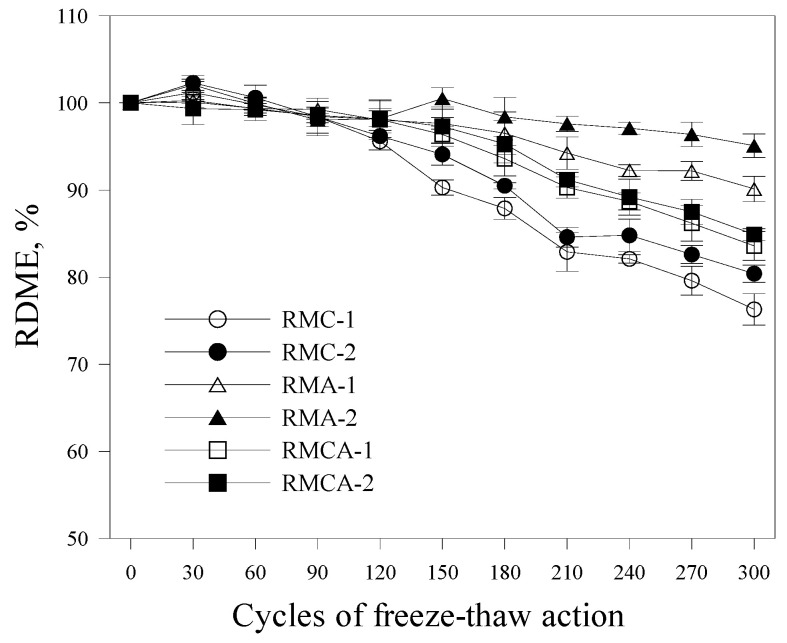
RDME of RM exposed to the freeze–thaw action.

As shown in Figure 9, after 300 cycles, RMA mortars retain RDME values above 90% (90.1% for RMA-1 and 95.1% for RMA-2), indicating superior freeze–thaw resistance compared with those of RMCA and RMC mortars. Conversely, the RDME of RMC mortars drop below 80% after 300 cycles, indicating comparatively poor resistance to freeze–thaw deterioration.

**Figure 10:**
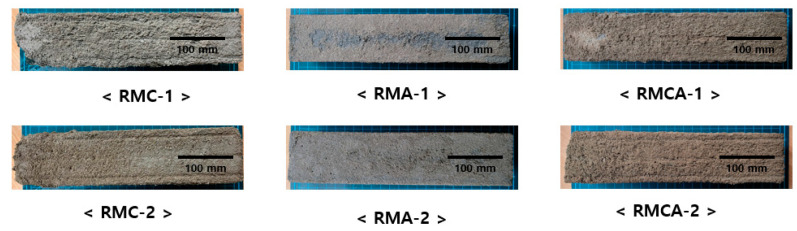
Visual appearance of RM exposed to 300 cycles of freeze–thaw action.

The visual appearance of mortars after 300 cycles is shown in Figure 10, which indicates that RMC-1 and RMC-2 specimens exhibit severe surface scaling, with evident spalling at edges and noticeable surface softening. In contrast, RMA mortars remain relatively intact, displaying only minor surface cracking and minimal deterioration. RMCA mortars (RMCA-1 and RMCA-2) experienced more significant surface degradation compared with RMA mortars, characterized primarily by surface scaling.

These findings indicate that ACA mortars provide the highest resistance to freeze–thaw action. Accordingly, RMs incorporating ACA are expected to be effective as rapid repair materials for concrete structures in cold regions.

### 3.6. XRD Analysis

**Figure 11:**
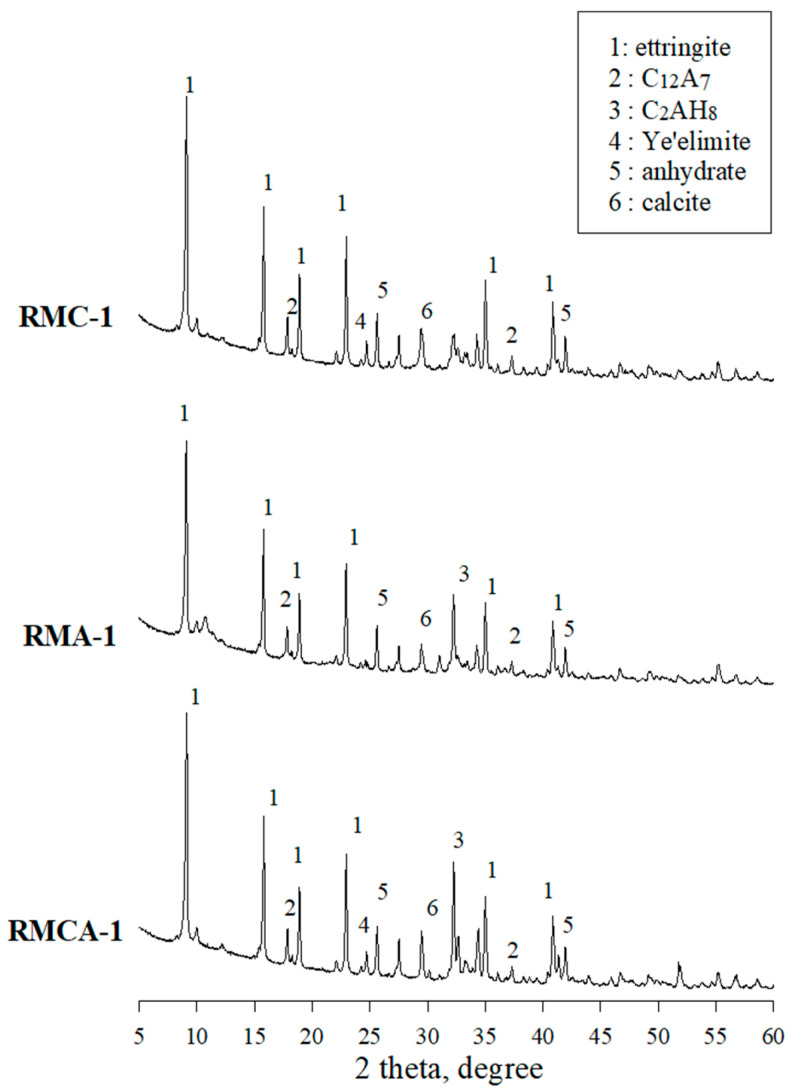
XRD patterns of paste sample after 28 days.

The XRD patterns of paste samples cured for 28 days are shown in Figure 11, revealing that the hydration products vary depending on the binder type.

In the RMC-1, RMA-1, and RMCA-1 samples incorporating CSA and ACA, strong peaks corresponding to ettringite are observed. Furthermore, peaks corresponding to C_12_A_7_ and C_2_AH_8_ are detected, attributed to the combined use of OPC and aluminate-based binders [12,14].

For the RMC-1 sample prepared with CSA, the XRD pattern validates the formation of substantial amounts of ettringite through the hydration of ye’elimite (3CaO_3_·Al_2_O_3_·CaSO_4_), consistent with the reactions expressed in Equations (3) and (4) [2].

3CAO·3Al_2_O_3_·CaSO_4_ + 2CaSO_4_ + 34H_2_O → 3CaO·Al_2_O_3_·3CaSO_4_·32H_2_O + 4Al(OH)_3_ (3)

3CAO·3Al_2_O_3_·CaSO_4_ + 8CaSO_4_ + 6Ca(OH)_2_ + 34H_2_O → 3CaO·Al_2_O_3_·3CaSO_4_·32H_2_O (4)

Distinct C_2_AH_8_ peaks are observed in the RMA-1 and RMCA-1 samples containing ACA. This phenomenon is attributed to the reaction of C_12_A_7_, the principal component of ACA, which promotes the consumption of portlandite during hydration and results in the formation of C_2_AH_8_ as the predominant product [12].

### 3.7. SEM Observations

**Figure 12:**
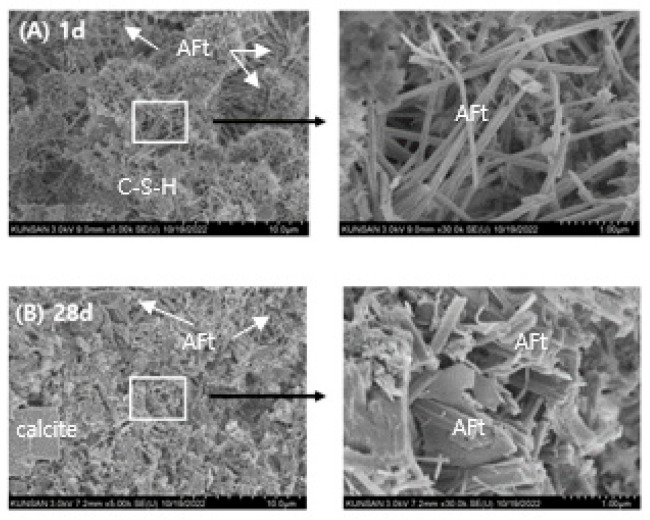
SEM images of RMC-1 sample; (**A**): 1 d, (**B**): 28 d. Scale bar: 10.0 μm (**left**), 1.0 μm (**right**).

**Figure 13:**
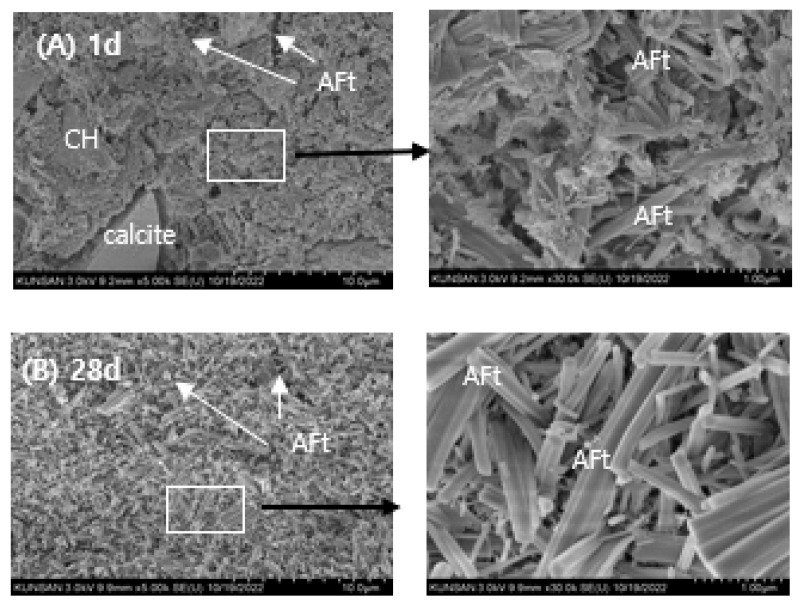
SEM images of RMA-1 sample; (**A**): 1 d, (**B**): 28 d. Scale bar: 10.0 μm (**left**), 1.0 μm (**right**).

**Figure 14:**
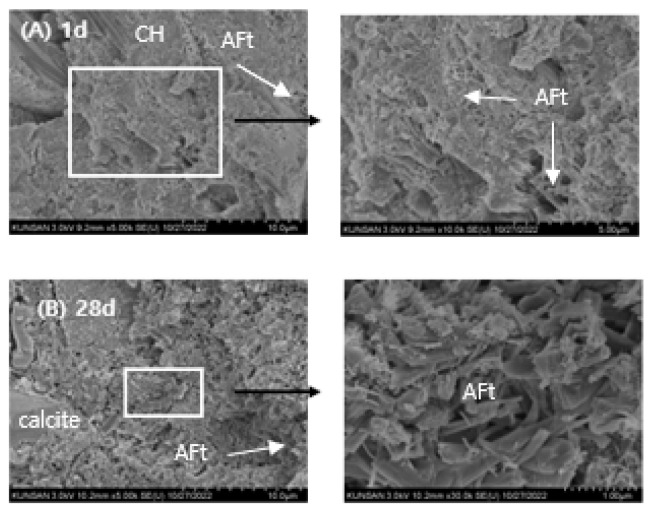
SEM images of RMCA-1 sample; (**A**): 1 d, (**B**): 28 d. Scale bar: 10.0 μm (**left**), 1.0 μm (**right**).

The microstructural evolution of RMC-1, RMA-1, and RMCA-1 pastes at 1 and 28 days are shown in Figure 12, Figure 13 and Figure 14.

The SEM images of the RMC-1 paste are shown in Figure 12. After 1 day of curing (Figure 12A), numerous long, needle-like AFt crystals are observed within the pore structure. Minor quantities of portlandite and C–S–H, products of OPC hydration, are also detected, accompanied by a high density of micro-pores. AFm phases are not detected at this stage. After 28 days of curing (Figure 12B), both needle-like and subhedral AFt crystals are prominent, whereas portlandite is scarcely observed.

The SEM images of the RMA-1 paste prepared with ACA as the primary binder are shown in Figure 13. After 1 day of curing (Figure 13A), the microstructure appears denser compared with RMC-1, with a reduced number of micro-pores. Both subhedral and needle-like AFt crystals are distributed throughout the sample. After 28 days of curing (Figure 13B), subhedral AFt crystals become the dominant feature.

The SEM images of RMCA-1 are shown in Figure 14. At the first and 28th days, the microstructure is comparatively less dense, with numerous pores and cracks. Abundant hexagonal, plate-like portlandite crystals are also observed. Consistent with the XRD results (Figure 11), ettringite is identified as the primary hydration product in RMCA-1.

These microstructural variations in hydration products are considered to significantly influence the mechanical and durability performance of the RMs.

## 4. Conclusions

This study experimentally evaluated the mechanical performance and microstructural characteristics of RMs incorporating aluminate-based binders. The conclusions are as follows:

1. The initial flow values of RMA mortars were relatively high; however, they demonstrated a more pronounced loss of fluidity over time. Overall, fluidity decreased as the dosage of aluminate-based binders increased.
2. The setting time was reduced by increasing the amount of CSA and ACA. The setting time of RMA mortars was approximately 50% shorter than that of RMC mortars, primarily attributed to the hydration of C_12_A_7_, which represents the primary component of ACA.
3. The compressive and flexural strength results indicated that the strength development of ACA mortars was superior to that of CSA mortars from the earliest ages. For example, the compressive strength of RMA-2 after 28 days of curing was significantly higher by 36.7% compared with that of RMC-2, with the difference further increasing to 74.1% after 1 day of curing. A similar trend was observed in flexural strength.
4. Higher contents of aluminate-based binders resulted in increased UPV values. The incorporation of ACA had a particularly positive effect on UPV; at the first and 28th days, the UPVs of RMA-2 were 3.99 and 5.56 km/s, which were approximately 35% and 48% higher than those of RMC-2, respectively.
5. In freeze–thaw resistance tests, RMA mortars maintained relative RDME values above 90%, whereas those of RMC mortars fell below 80%, validating the significance of ACA in enhancing durability. After 300 cycles, visual inspection revealed that RMA specimens remained relatively intact, displaying only minor surface cracks. In contrast, RMC specimens demonstrated pronounced surface scaling, spalling, and softening.
6. XRD analysis revealed that RMC samples generated significant amounts of ettringite through the hydration of ye’elimite, whereas ACA samples primarily generated C_2_AH_8_, resulting from C_12_A_7_ hydration. These distinct hydration products are believed to significantly impact the mechanical properties and durability of the mortars.

Based on the experimental results, ACA-based mortar exhibited rapid setting, excellent mechanical properties, and durability (frost resistance). This was mainly attributed to the formation of C_2_AH_8_ and AFt during ACA hydration, as revealed by XRD and SEM analysis, and can be considered to have promoted densification of the mortar microstructure especially from an early stage of curing. This suggests that ACA is an appropriate repair material for deteriorated structures in cold regions that require rapid setting and high-early strength, while CSA-based repair materials are applicable to the repair of concrete structures exposed to common damage factors.

## Figures and Tables

**Table 1:** Chemical composition of binders (%).

	SiO_2_	Al_2_O_3_	Fe_2_O_3_	CaO	MgO	SO_3_	TiO_2_	LOI
OPC	19.8	4.8	3.1	61.5	2.9	2.8	0.3	1.3
CSA	9.1	28.0	3.1	44.3	2.5	10.1	1.0	0.70
ACA	3.8	42.8	0.8	48.4	1.2	0.2	2.2	0.3
AG	3.3	1.5	0.2	42.4	0.3	51.9	-	-

**Table 2:** Mix proportions of RM.

Mixes	w/b (%)	Binders (g)				Fines (g)
		OPC	CSA	ACA	AG	
RMC-1	57	17.5	8.75	-	8.75	70
RMC-2		10.5	14.0	-	10.5	
RMA-1		17.5	-	8.75	8.75	
RMA-2		10.5	-	14.0	10.5	
RMCA-1		17.5	4.38	4.38	8.75	
RMCA-2		10.5	7	7	10.5	

## Data Availability

The original contributions presented in the study are included in the article. Further inquiries can be directed to the corresponding author.

## Abbreviations

The following abbreviations are used in this manuscript:

RM	Repair mortars
CSA	Calcium sulfoaluminate
AG	Anhydrite gypsum
UPV	Ultrasonic pulse velocity
RDME	Relative dynamic modulus of elasticity
OPC	Ordinary Portland cement
ACA	Amorphous calcium aluminate
XRD	X-ray diffraction
SEM	Scanning electron microscopy
